# Growth Associated Protein 43 Is Expressed in Skeletal Muscle Fibers and Is Localized in Proximity of Mitochondria and Calcium Release Units

**DOI:** 10.1371/journal.pone.0053267

**Published:** 2013-01-07

**Authors:** Simone Guarnieri, Caterina Morabito, Cecilia Paolini, Simona Boncompagni, Raffaele Pilla, Giorgio Fanò-Illic, Maria A. Mariggiò

**Affiliations:** 1 Department of Neuroscience and Imaging (DNI), University G. d’Annunzio, Chieti, Italy; 2 Center for Research on Ageing (CeSI), University G. d’Annunzio, Chieti, Italy; 3 Interuniversitary Institute of Myology (IIM), University G. d’Annunzio, Chieti, Italy; Mayo Clinic, United States of America

## Abstract

The neuronal Growth Associated Protein 43 (GAP43), also known as B-50 or neuromodulin, is involved in mechanisms controlling pathfinding and branching of neurons during development and regeneration. For many years this protein was classified as *neuron-specific*, but recent evidences suggest that a) GAP43 is expressed in the nervous system not only in neurons, but also in glial cells, and b) probably it is present also in other tissues. In particular, its expression was revealed in muscles from patients affected by various myopathies, indicating that GAP43 can no-longer considered only as a neuron-specific molecule. We have investigated the expression and subcellular localization of GAP43 in mouse satellite cells, myotubes, and adult muscle (extensor digitorum longus or EDL) using Western blotting, immuno-fluorescence combined to confocal microscopy and electron microscopy. Our *in vitro* results indicated that GAP43 is indeed expressed in both myoblasts and differentiating myotubes, and its cellular localization changes dramatically during maturation: in myoblasts the localization appeared to be mostly nuclear, whereas with differentiation the protein started to display a sarcomeric-like pattern. In adult fibers, GAP43 expression was evident with the protein labeling forming (in longitudinal views) a double cross striation reminiscent of the staining pattern of other organelles, such as calcium release units (CRUs) and mitochondria. Double immuno-staining and experiments done in EDL muscles fixed at different sarcomere lengths, allowed us to determine the localization, from the sarcomere Z-line, of GAP43 positive *foci*, falling between that of CRUs and of mitochondria. Staining of cross sections added a detail to the puzzle: GAP43 labeling formed a reticular pattern surrounding individual myofibrils, but excluding contractile elements. This work leads the way to further investigation about the possible physiological and structural role of GAP43 protein in adult fiber function and disease.

## Introduction

The neuronal Growth Associated Protein 43 (GAP43), also known as neuromodulin, was first isolated about 30 years ago from synaptosomal plasma membranes of rat brain and identified as a B-50 phosphoprotein band [1], [2]. This protein is highly conserved, expressed in different species, and regulated by post-translational mechanisms, which modulate its intracellular functions [3].

It is widely accepted that GAP43 (or B-50) plays key roles in neurons: it is involved in mechanisms controlling pathfinding and branching during development and regeneration [4], [5], and may contribute to pre-synaptic membrane changes modulating neurotransmitter release, endocytosis, synaptic vesicle recycling, etc. [3], [6], [7]. The importance of GAP43, in general, is demonstrated by the fact that deletion of the GAP43 gene in mice [8]. Knockout of GAP43 in mice results in considerable reduction in dimensions of cerebellum, possibly due to the impaired neuronal branching and pathfinding, and finally leads to high rate mice lethality (90–95%) within 2 days after birth [8]–[10]. These data are supported by data obtained in transgenic mice: overexpression of GAP43 leads to the spontaneous formation of new synapses and enhanced sprouting after injury [11]. Whereas transgenic mice expressing a mutant GAP43, that cannot be phosphorylated by protein kinase C (PKC), presented a reduced sprout-promoting activity, demonstrating that phosphorilation is crucial for GAP43 effect on neurite outgrowth [11].

Even though GAP43 was classified as neuron-specific protein, increasing evidences indicate that GAP43 is not confined to neurons and that, likely, its expression is not confined to the nervous system.

This protein was indeed found in glial cells, as Schwann cells, during the degenerative process of peripheral nerves [12]–[15]. In support of these findings Ma and Collaborators reported that, during the functional recovery of neuromuscular junctions, GAP43 was up-regulated, speculating that it could be due to nerve sprouting and Schwann cells activation [16], [17].

Evidences that GAP43 expression is not confined to the nervous system, were collected in embryonic chicken limb and in human skeletal muscle biopsies [18]–[20]. Heuss and colleagues reported the expression of B-50 in some pathological skeletal muscle hypothesizing its involvment in muscle diseases and concluding that “*B-50 can no-longer be regarded as a neuron-specific molecule*” [18], [21]. However, in those studies no demonstrable B-50 staining was found in normal adult skeletal muscle and to date there is no evidence of GAP43 expression in adult muscle.

In the present work we studied the expression and intracellular localization of GAP43 in mouse satellite cells (myoblasts representing the regenerative system of skeletal muscle), myotubes and adult skeletal fibers from mouse extensor digitorum longus (EDL) muscle, using Western blotting, immuno-fluorescence combined to confocal microscopy and electron microscopy (EM).

To determine the subcellular distribution of GAP43 it must be considered that adult skeletal muscle fibers are characterized by: a) an extremely high degree of intracellular organizations and b) specific disposition of organelles such as Ca^2+^ release units (CRUs) and mitochondria in respect to sarcomeric striation [22].

Our results show for the first time that GAP43 is expressed in the mouse muscle regenerative system and also in healthy adult fibers in which its subcellular localization presents a double sarcomeric distribution, resembling the pattern of triadic proteins and of mitochondria, but with a slightly different spacing.

This work leads the way to further investigation of the possible mechanisms in which GAP43 could be involved and of its physiological and structural role in muscle cells and adult fibers.

## Materials and Methods

### Ethics Statement

The care and use of wild type (WT) mice (C57BL/6, Charles River Laboratories Italia s.r.l., Calco, Italy) strictly followed “*The Guiding Principles for the Care and Use of Animals*”, in accordance with the principles of the Declaration of Helsinki and with the European Community Council (86/609/CEE) and the Italian Government law on the protection of animals for experimental procedures in research laboratory (92/116).

Mice were housed in a facility c/o D’Annunzio Foundation Ce.S.I. (Center for Research on Ageing, Chieti, Italy) and sacrificed by cervical dislocation as approved by the local University Committee on Animal Resources (Comitato di Etica Interateneo per la Sperimentazione Animale - CEISA Università “G.d’Annunzio” Chieti-Pescara – Università di Teramo prot. n. 15/2011/CEISA/COM).

### Chemicals and Materials

Unless otherwise indicated, cell culture media, sera, antibiotics were obtained from Invitrogen (S. Giuliano Milanese, Italy), cell culture dishware from Becton Dickinson Falcon™ (Sacco Srl, Cadorago, Italy), and reagents and standards from Sigma-Aldrich (Milan, Italy).

### Animal Housing and Skeletal Muscle Fiber Isolation

Mice were sacrificed by cervical dislocation and the extensor digitorum longus (EDL) was dissected manually under stereoscopic observation, from about 4 month old mice, and fixed either in a 4% paraformaldehyde (Sigma-Aldrich) solution in PBS for immuno-fluorescence or in 3.5% glutaraldehyde in 0.1 M sodium cacodylate buffer (pH 7.2) for electron microscopy, or washed in PBS to be processed for Western blot procedure.

### Cell Cultures

#### a. Satellite cells

WT adult male mice were killed by cervical dislocation according to the recommendations of local University Committee on Animal Resources (15/2011/CEISA/COM). After sterile dissection, whole muscle was manually shredded and digested for 30 min at 37°C with 1 mg/ml Collagenase/Dispase (Roche Diagnostics, Mannheim, Germany) in PBS. After centrifugation (150×g for 5 min), the pellet was digested for 15 min at 37°C with 0.1 mg/ml Collagenase Type II (Sigma-Aldrich) in PBS. The enzymatic reaction was blocked adding cellular growth medium (high-glucose Dulbecco’s modified Eagle’s medium, DMEM, supplemented with 20% horse serum/3% chick embryonic extract, 4 mM L-glutammine and 100 UI ml^−1^–100 µg ml^−1^ penicillin-streptomycin), then the suspension, after filtering using 40 micron cell strainer filter (Falcon), was centrifuged at 200×g for 15 min, The pellet, containing the cells, was resuspended in growth medium, and the cells were pre-plated twice for 1 h, in order to remove the fibroblasts. The satellite cells were then plated on collagen coated dishes and grown in growth medium. After 5 days of culture, satellite cells were shifted in differentiation medium (DMEM supplemented with 5% horse serum/3% chick embryonic extract 1% penicillin/streptomycin and 4 mM L-glutamine) [23].

#### b. Cell lines

C2C12 (ATCC, MD, USA), L6C5 (a kind gift from Dr A. Musarò, University La Sapienza, Rome, Italy) [24] cell lines were routinely maintained at 37°C with 5% CO_2_ humidified atmosphere in a CO_2_ incubator. The cells were expanded in DMEM supplemented with 20% fetal calf serum (FCS), 2 mM L-glutamine and 100 UI ml^−1^–100 µg ml^−1^ penicillin-streptomycin. The differentiated phenotype (myotubes resulting from the fusion of myoblasts) was induced when cells reached about 70% confluence by replacing DMEM with 2% heat-inactivated horse serum instead 20% FCS.

### Western Blot Analysis

Protein extracts for Western blot, were isolated from cells, muscle fibers and whole brain. The cells were rapidly washed on ice with pre-cooled PBS and sonicated in a lysis buffer (10 mM Tris–HCl, 5 mM EDTA, 7.5 mM MgCl_2_, 0.1 mM phenylmethylsulfonyl fluoride, 0.01 mg/ml leupeptin, 0.005 mg/ml pepstatin A, 0.01 mg/ml benzamidine, pH 7.4). The sonicated cells were centrifuged for 10 min (1000×g) at 4°C, supernatants were collected, and protein concentration was determined using the Bio-Rad protein assay (Bio-Rad Laboratories) [25]. Isolated muscle fibers were washed in PBS and homogenized in the same lysis buffer using a Polytron tissue disrupter (Janke and Kunkel, Germany), at high speed (three 10 s pulses) on ice. C57BL/6 whole brain proteins were obtained from tissue homogenization in ice-cold lysis buffer using a Dounce tight-fitting homogenizer. The homogenate was centrifuged at 1000 × g at 4°C, for 5 min to remove cell debris; protein concentration was determined as above reported.

Samples, containing 50 or 2.5 µg of protein, for muscle or brain respectively, were suspended in Laemmli buffer (8% (w/v) SDS, 10% (v/v) glycerol, 5% (v/v) β-mercaptoethanol, 25 mM Tris–HCl, pH 6.5, and 0.003% (w/v) bromophenol blue), boiled for 5 min, and separated by SDS–PAGE on a 10% (w/v) homogeneous slab gel. Proteins were electroblotted onto a hydrophobic polyvinylidene difluoride membrane (Immobilon-P membrane; Millipore, Bedford, MA, USA). Membranes were blocked in TBS-T (Tris-buffered saline with 0.1% (v/v) Tween 20) containing 5% (w/v) fat-free milk and then incubated with the primary antibodies anti-GAP43. The membranes were then incubated with horseradish peroxidase-conjugated antiIgG and detected by chemiluminescence (ECL Plus; GE–Amersham, Little Chalfont, UK).

### Immuno-staining of Undifferentiated Cells, Myotubes and Muscle Fibers

Undifferentiated or differentiated cells, plated onto 12-mm glass coverslips, and small bundles of fibers were fixed in a 4% paraformaldehyde solution in PBS for 30 min. Cross sections were obatined embedding the EDL muscle in 7% agarose in PBS. Agarose blocks were cut in 90-µm sections with a vibratome (Leica VT1000A, Leica Microsystems srl, Milan Italy). All samples were washed three times in PBS and permeabilized with a 0.2% Triton X-100 solution for 10 min, then incubated in blocking buffer (PBS plus 10% goat serum, for 1 h at room temperature, RT) followed by an overnight incubation at 4°C with primary antibody (mouse monoclonal anti-GAP43, mGAP or rabbit polyclonal anti-GAP43, HPA-GAP43, dil. 1∶500 Sigma-Aldrich; monoclonal anti-α-actinin sarcomeric dil. 1∶500 Sigma-Aldrich; rabbit affinity-purified TRN6 antibody raised against residues 146–160 of mouse triadin, dil. 1∶200 kindly gifted by Dr L.A. Jones; rabbit polyclonal anti-RyR1 (no 5), dil. 1∶200 gift of Dr S. Fleischer [26]; mouse monoclonal anti-RyR1/RyR3 (34C antibody), dil. 1∶20 (Developmental Studies Hybridoma Bank – University of Iowa) [27]; rabbit polyclonal anti-mitochondrial preprotein translocases of the outer membrane,TOM20, Santa Cruz Biotechnology Inc), the primary antibodies were revealed by a 2 h incubation with the specific secondary antibodies (goat anti-mouse Alexa Fluor-488 and goat anti-rabbit Alexa Fluor-568 dil. 1∶200, Invitrogen) at RT.

Images were acquired using a Zeiss LSM510 META system (Jena, Germany) equipped with an Zeiss Axiovert 200 inverted microscope and a Plan Neofluar oil-immersion objective (100X/1.3 NA). Negative controls for each immuno-staining assay were performed by sample immuno-labeling with only secondary antibodies. Data of fluorescence image profiles were obtained from LSM 3.0 image analysis software by Zeiss. Co-localizations were determined by ImageJ and Image Correlator Plus Plug-in® (NIH Image, Bethesda, MD) and were quantified by Pearson’s correlation coefficient; values of 0 indicated no co-localization, and values of 1 indicated complete co-localization [28].

### Electron Microscopy

C57Bl/6 between 3 and 4 months of age were sacrificed by cervical dislocation. EDL muscles were dissected, pinned to a Sylgard dish (Dow Corning) at resting length (approximately 10 mm long from tendon to tendon) and stretched length (approximately 13 mm long from tendon to tendon) and fixed with 3.5% glutaraldehyde in 0.1 M sodium cacodylate buffer (pH 7.2) at RT. Small bundles of fixed muscle were post-fixed in 2% OsO_4_ in the same sodium cacodylate buffer for 1–2 h at 4°C and block-stained in saturated uranyl acetate. After dehydration, the specimens were embedded in an epoxy resin (Epon 812). Thin longitudinal sections (∼40 nm) were cut in an ultramicrotome Leica Ultracut R (Leica Microsystem, Vienna, Austria) using a Diatome diamond knife (Diatome Ltd., CH-2501, Biel, Switzerland) and stained in 4% uranyl acetate and lead citrate solutions. The stained sections were examined with a FP 505 Morgagni Series 268D electron microscope (Philips, Brno, Czech Republic) at 60 kV equipped with a Megaview III digital camera and Soft Imaging System (Munster, Germany).

### Morphometric Analyses

#### a. Fluorescence immuno-staining

Morphometric analyses were carried out on immuno-labeled fibers from EDL muscle fixed with 4% paraformaldehyde solution in PBS (30 min) at ∼9 and 13 mm length (resting or stretched length). In the GAP43 stained samples, the maximum and minimum distances between two striation (named intra-sarcomere and inter-sarcomere, respectively) were measured. The α-actinin staining was used for measuring the distance between two Z-lines. Measurements were performed on 4 different EDLs for each condition, on post-acquisition images using a Zeiss LSM 5 Image Examiner software.

#### b. Electron microscopy

The following distances were measured in non-overlapping micrographs taken randomly at 14,000x in 10 different fibers from rest and stretched EDLs, as described in immuno-fluorescence:

- i.sarcomere length taken from Z-line to Z-line;
- intra- and inter-sarcomere T-tubule distances taken from triad T-tubule center to triad T-tubule center;
- intra and inter-sarcomere mitochondria distances taken from I band mitochondria center to I band mitochondria center.

Measurements were performed using the AnalySIS software provided with the EM digital camera.

## Results

### Expression of GAP43 in Skeletal Muscle Models

**Figure 1:**
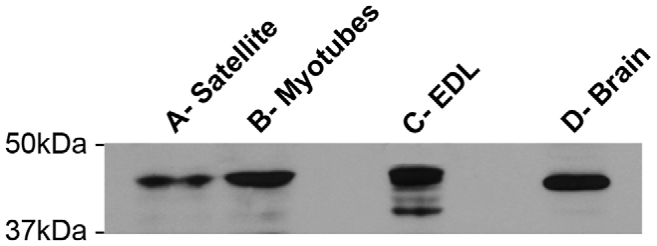
GAP43 is expressed at all stages of skeletal muscle maturation. Representative western blot of samples obtained from: A) proliferating myoblasts; B) differentiated myotubes; and C) adult extensor digitorum longus (EDL, 4 months of age). On the right, a preparation obtained from mouse brain (D- Brain) is used as positive control. All specimens (i.e. satellite cells, myotubes, EDL and brain) show the same immuno-reaction at ∼43KDa, when probed with the mGAP43 antibody.

The presence of GAP43 was verified in different sample preparations obtained from: proliferating mouse satellite cells, myotubes, and EDL muscle fibers, as well as from mouse brain used as positive control (Figure 1). The Western blotting revealed a specific immuno-reactive band at 43KDa when the membrane was probed with mouse monoclonal anti-GAP43 antibody (mGAP43) indicating the presence of the protein in muscle preparations as well as in brain extracts. In particular, the GAP43 expression levels resulted lower in muscle samples in respect to the brain one, considering that 50 µg total protein, for muscle samples and 2.5 µg of brain extract were subjected to electrophoresis (see Matherials and Methods). Similar results were obtained also testing the blot membranes with a rabbit polyclonal anti-GAP43 antibody (HPA-GAP43, see Figure. S1A).

### During in vitro Cell Differentiation GAP43 Acquires Sarcomeric Localization

**Figure 2:**
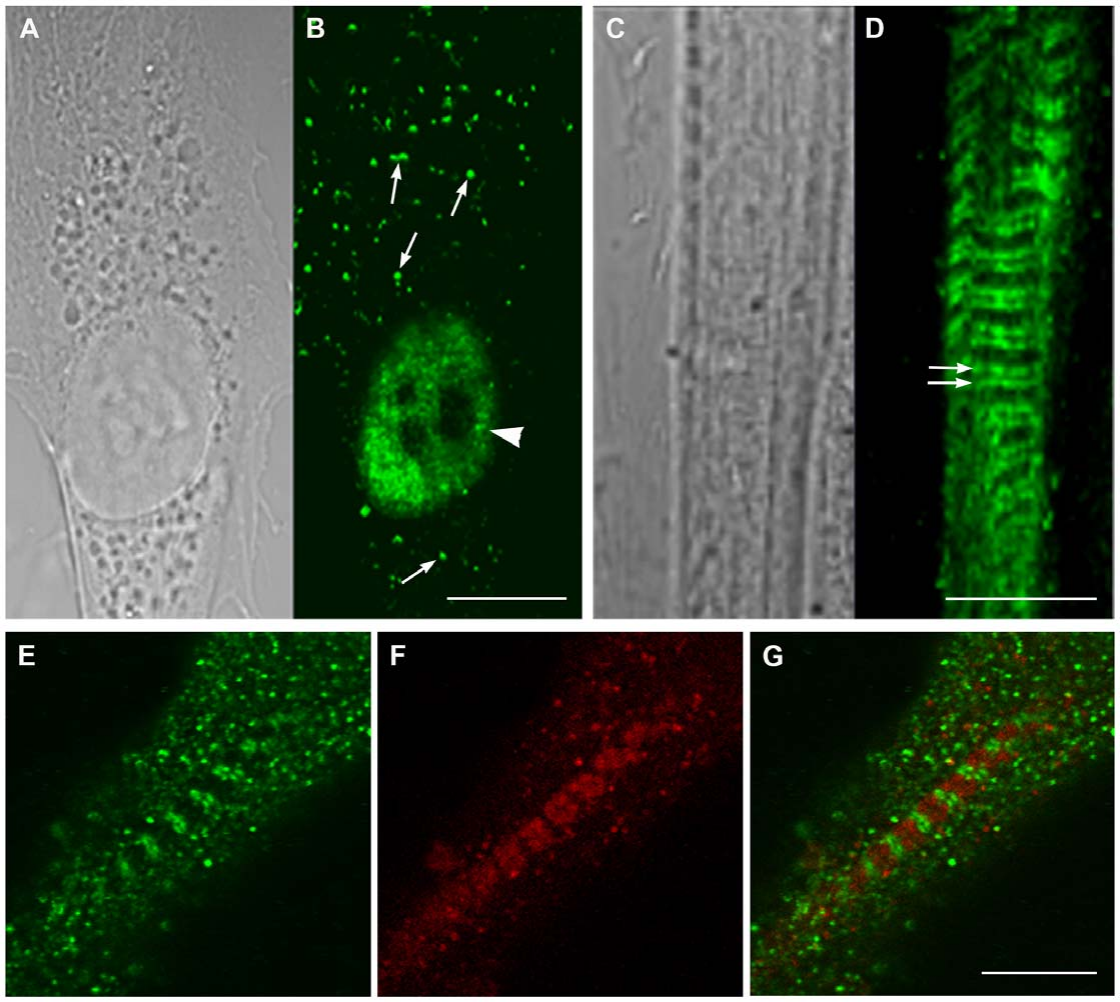
Localization of GAP43 changes in myoblasts and myotubes from nuclear to sarcomeric. Light and confocal microscopy of proliferating myoblasts (A) and differentiated myotubes (C) stained with the mGAP43 antibody (B and D) reveal changes in localization during the differentiation process. A-B) GAP43 is mainly localized in the nucleus in un-differentiated cells (arrowhead), even if bright spots are also visible in the cytoplasm. C-D) The nuclear localization typical of proliferating myoblasts disappears with differentiation, i.e. it is replaced by a striated pattern (double arrows). E-G) Double labeling of myotubes with anti-GAP43 (green, E) and anti-triadin (red, F) antibodies shows that GAP43 double cross striation does not superimpose with triadin, a protein marking the position of CRUs (G, merged image). Bars: 10 µm.

The cellular localization of GAP43 was assayed both in proliferating mouse satellite cells as well as myotubes derived from satellite cell fusion. Results from immuno-fluorescence combined to confocal microscopy revealed that the protein cellular localization changed dramatically after maturation: in myoblasts the localization appeared to be mostly nuclear, whereas in myotubes the protein clearly localized in a regular sarcomeric-like pattern (Figure 2 B, D, E. G). A similar pattern was observed also in other skeletal muscle cell lines such as the mouse C2C12, or the rat L6E9 cells. The protein appeared localized mostly in the nuclei in C2C12 and L6E9 myoblasts, whereas it localized in regular pattern in the cytoplasm in differentiated C2C12 or L6E9 (Figure S2). The sarcomeric localization became evident in triadin/GAP43 double labeled samples from satellite-derived differentiating myotubes, where GAP43 moved from an unordered spotted cytoplasmic localization to regular double strikes closed but not co-localized with triadin (Figure 2 E, F, G).

### In Adult Skeletal Muscle GAP43 is Located Close to CRUs and Mitochondria

**Figure 3:**
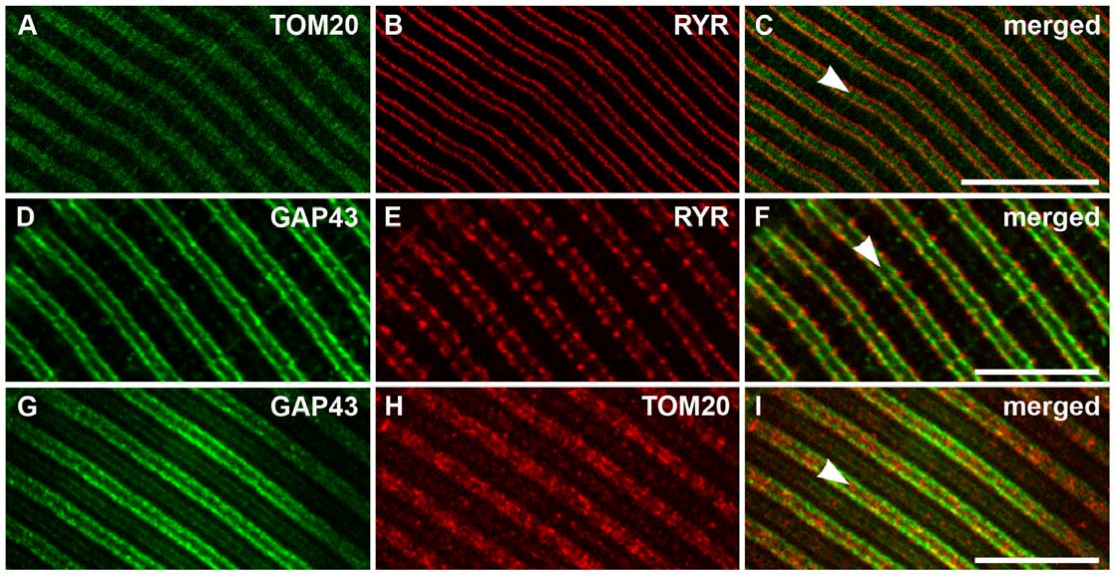
In adult fibers, GAP43 is localized between Z-lines and CRUs, close to the band occupied by mitochondria. A-C) In adult EDL fibers, both CRUs (marked with an anti-RYR antiboby) and mitochondria (marked with an anti-TOM20 antibody) staining form double rows on both sides of the Z-lines (the position of which is marked by arrowheads in merged figures), with RYR flanking TOM20 staining. D-I) Also GAP43 staining produces a double cross-striation (D and G), which seems to be partially co-localized with that of RYR (F) but outside that of mitochondria (I). Bar: 10 µm.

Considering the observation of the ordered and regular placing of GAP43 in myotubes and the organization of the functional organelles (i.e. CRUs and mitochondria) in skeletal fibers, we studied the localization of the GAP43 in adult mouse (3–4 months) EDL muscle. Fibers were co-stained using mGAP43 antibody and rabbit anti-ryanodine receptor antibody, to mark the position of ryanodine receptors, or anti-translocase of outer mitochondrial membranes antibody, to recognize the mitochondria. The results showed that also in EDL, GAP43 drew a regular double streaks crossing the fiber (Figure 3 D and G), resembling the same pattern observed in myotubes (Figure 2D). A similar arrangement, even if with a punctuate pattern, was also evident using the HPA-GAP43 antibody (Figure S1B). The double immuno-labeling approach allowed to define the relationship between GAP43 and other functional structures of skeletal muscle fiber. The co-immunostaining of mitochondria and RYR (Figure 3, A, B, C), confirmed the localization of these structures already described [22], with RYR strikes enclosing mitochondria with no points of co-localization. The co-staining with GAP43 revealed that RYR appeared outside the anti-GAP43 positive double striations and partially co-localized (Figure 3F;). Mitochondria localization, revealed by anti-TOM20 antibody, appeared to describe a double rows (Figure 3B and H), as already described [22]. Merged image with GAP43 positive fluorescence showed that mitochondria were localized inside the double strand defined by GAP43 that in some points appeared to be co-localized (Figure 3I). These results, collectively suggested that GAP43 localized between CRUs and mitochondria.

### GAP43 is Localized Around the Myofibrils and Rarely Co-localized with Mitochondria

**Figure 4:**
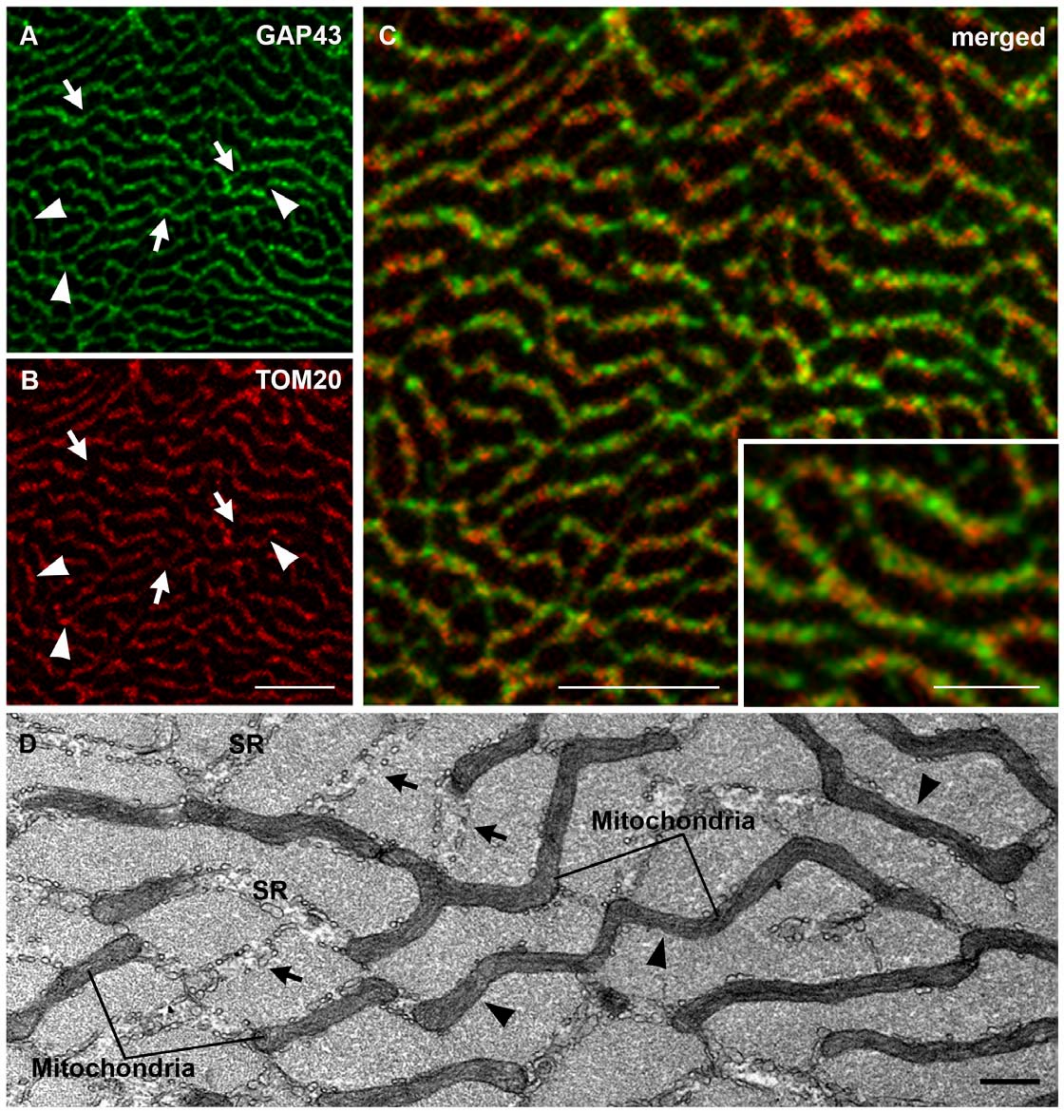
GAP43 is localized around the myofibrils and alternates to mitochondria. A-C) Immuno-fluorescence of transverse sections stained with anti-GAP43 antibody, shows a reticular pattern surrounding myofibrils, quite similar to that formed by mitochondria (marked by TOM20). In some points red and green foci are contiguous, clearly distinct (inset in C). In A and B arrowheads indicate only TOM20 (red) staining, while arrows only GAP43 (green) staining. D) Electron micrograph of an EDL muscle in cross section. Both mitochondria and sarcoplasmic reticulum (SR) encircling myofibrils forming a network. In D arrowheads indicate only mitochondria presence, while arrows only SR. Bars: A-D,10 µm; D, 0.500 µm.

**Figure 5:**
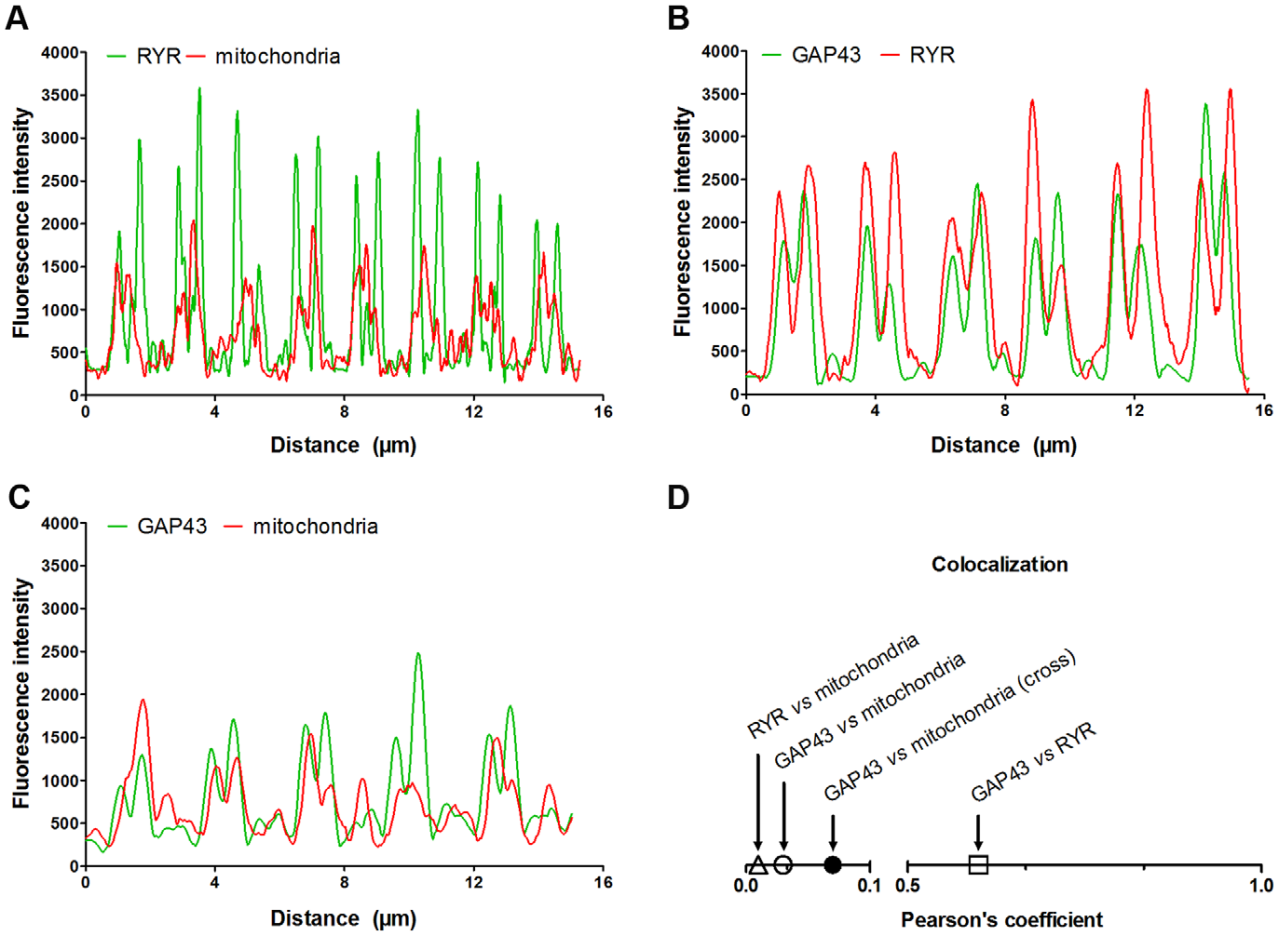
Fluorescence image profile and co-localization analyses. Graphs represent fluorescence intensity profiles calculated on images obtained from samples co-immunostained for; A) RYR and mitochondria; B); GAP43 and RYR; and C) GAP43 and mitochondria. The RyR fluorescence peak appears closer to that of GAP43 than mitochondria’s one in which the line profile is slight shifted in respect to that of GAP43 (B-C). D) Graph of the degree of co-localization calculated by Pearson’s coefficient in samples stained as in A-C, shows the different degree of co-localization between GAP43 and the other structure/organelles around the Z-line (i.e. mitochondria and CRUs).

To obtain further information on GAP43 localization, immuno-fluorescence stainings of EDL cross sections were performed. The analysis of the confocal images showed that GAP43 was organized in a reticular pattern surrounding myofibrils (Figure 4A) in a way similar to that described by mitochondria (Figure 4B). It is important to highlight that even though mitochondria and GAP43 appeared to describe a similar localization pattern, i.e. running in parallel surrounding each myofibril, they very rarely co-localized (yellow color, Figure 3C). The fluorescence signal derived from GAP43 and mitochondria double staining did not overlap and where GAP43 is present, mitochondria are missing, and *vice versa* (arrows and arrowheads in Figure 4A and B). This result suggested that GAP43 and mitochondria are contiguous and clearly distinct. Previous studies have shown that mitochondria and sarcoplasmic reticulum (SR) in skeletal muscle fibers are in close proximity [29], [30]. Mitochondria are found mostly in correspondence of the I band, and are usually closely apposed to the SR, adjacent to CRUs or triads. In transversely oriented electron micrographs, this association, i.e. splitting of the two structures, was visible. SR and mitochondria clearly alternate and rarely co-localized (arrows and arrowheads in Figure 4D). Considering GAP43 disposition with respect to RYR and mitochondria in longitudinal section, and the specific pattern described by GAP43 staining in cross section, it is possible to hypothesize a GAP43 localization between mitochondria and RYR. The different localization of GAP43 and mitochondria/RYR is illustrated by a computed fluorescence intensity profile along a straight line through a single myofiber in a fluorescence image. Along the fiber, the maxima of the intensities of GAP43- and mitochondria/RYR-specific fluorescence did not overlap (Figure 5). According to the profile analysis, while mitochondria intensity peaks were entirely included within the RYR’s (Figure 5 A), GAP43 peaks appeared to be positioned between the two, but closer to RYR than to mitochondria, which showed the line profile slightly shifted (Figure 5 B and C). The results from calculation of the degree of co-localization, using the Pearson’s coefficient, confirmed the data obtained in the fluorescence intensity profile analysis (Figure 5 D).

### The Position of GAP43 is Specifically between CRUs and Mitochondria

**Figure 6:**
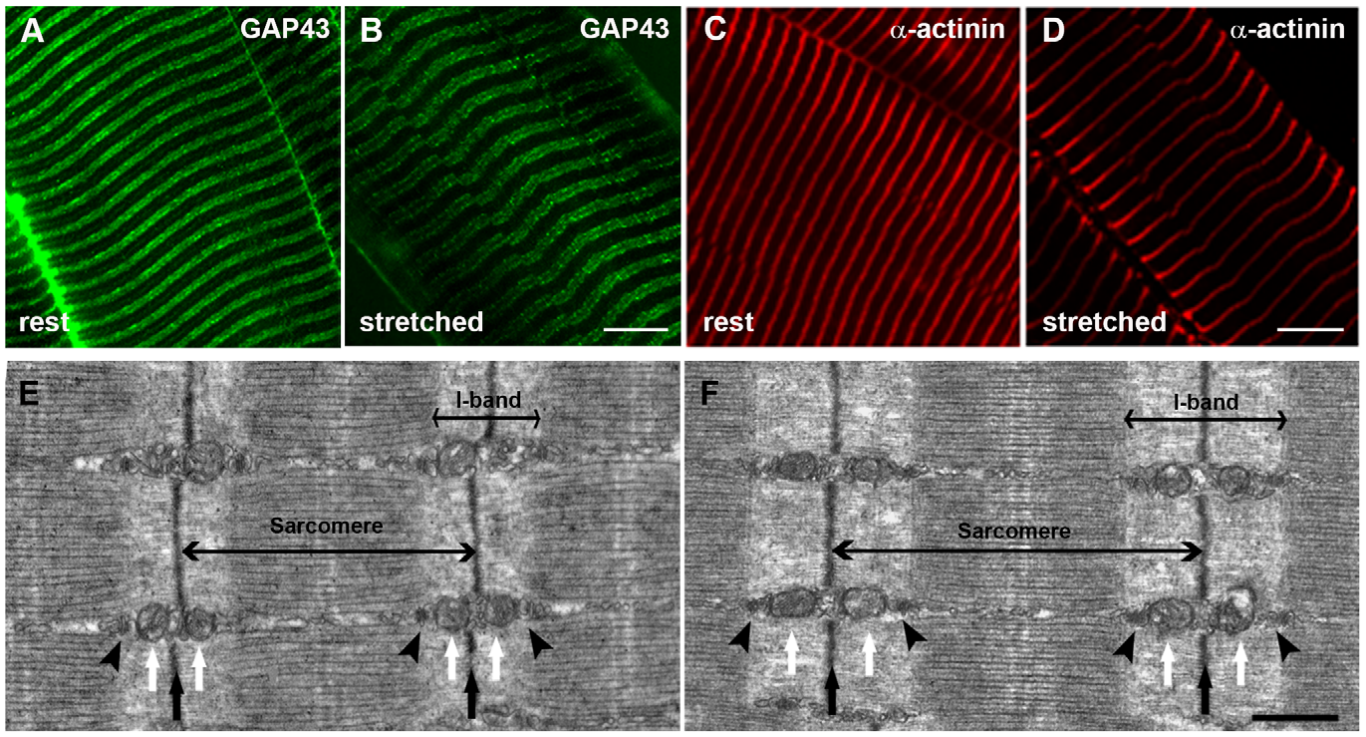
Immuno-fluorescence and electron microscopy (EM) of EDL fibers at different sarcomere lengths. Immuno-fluorescence with antibodies against GAP43 (A and B) and a-actinin (C and D), marking the position of Z-lines, and EM images (E and F) of EDL fibers prepared at two different length: rest (∼1.9µm) and stretched (∼2.5µm) were used to generate the data included in Tables I and II of Figure 7. Arrowheads in the EM micrographs (E and F) point to CRUs, white arrows point to mitochondria and black arrows mark the position of Z-disks. Bars: A-F, 10 µm; G-H, 0.500 µm.

**Figure 7:**
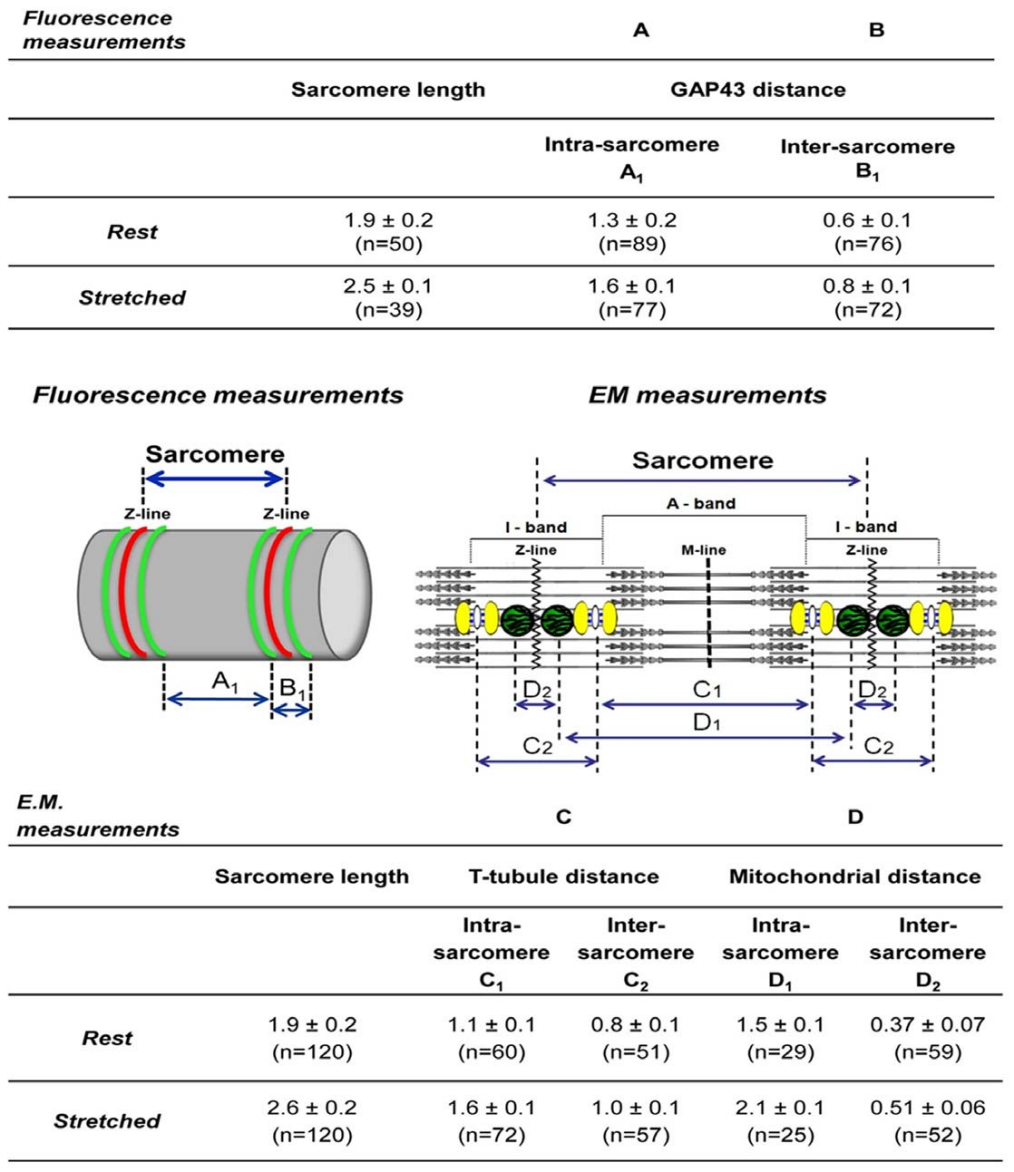
Quantitative analyses of images presented in **Figure 6**. Data in columns A and B (Fluorescence measurements) and in columns C and D (E.M measurements) contain respectively average measurements collected on immuno-fluorescence images (indicated by A1 and B1 in the left cartoon) and on electron microscopy micrographs (indicated by C1–C2 and D1–D2 in the right cartoon). Analysis of these data indicates that the position of GAP43 is between CRUs and mitochondria (see text for more details).

More details about the possible position of GAP43 in skeletal muscle fibers came from qualitative (Figure 6) and quantitative (Figure 7) analyses of EDL fibers fixed at two different sarcomere lengths both in immuno-fluorescence and EM.

Stretching of resting fibers by fixing EDL muscles at about 9 mm and 13 mm length (see Materials and Methods for more detail), did not change the specific GAP43 and α-actinin immuno-staining patterns (Figure 6, A, B, C, D). However, it is clearly noticeable an increase in the distance between the adjacent transverse lines. In EM longitudinal sections (Figure 6 E and F), the disposition of the two main intracellular organelles, CRUs and mitochondria, in respect to the sarcomere striation was clearly visible, highly organized and stereotyped as previously described [22]. CRUs are transversely oriented, located on both sides of the Z-line in proximity of the transition between A (dark) and I (pale) bands of the sarcomere (Figure 6 E and F, arrowheads). Mitochondria (Figure 6 E and F white arrows), where present, are usually placed next to CRUs on the side closer to the Z-line (Figure 6 E and F black arrows).

Quantitative analyses (Figure 7) were performed on samples described in Figure 6, measuring the intra- (within a single sarcomere) and inter- (across a single Z-line) sarcomere distances between two GAP43 striation in immuno-fluorescence images, at the two different sarcomere lengths. In parallel, intra- and inter-sarcomere distances between CRUs and between mitochondria were performed on electron-micrographs. Average data collected in these analyses were reported in Tables of Figure 7. The distance between GAP43 striations increased in stretched fibers (i.e. 2.5 µm sarcomere length) when compared to rest ones (i.e. 1.9 µm sarcomere length). In the same way, in EM micrographs, T-tubule distances and mitochondria distances increased. Comparing the data from immuno-fluorensce and EM, it was possible to hypothesize that GAP43 was located between CRUs and mitochondria. In fact, at rest length the average intra-sarcomere GAP43 distance was about 1.3 µm, between the average T-tubule distance (1.1 µm) and the average mitochondria distance (1.5 µm). Analyzing the inter-sarcomere data, GAP43 distance was 0.6 µm, which was intermediate between the mitochondria distances and T-tubule distances (0.8 µm and 0.4 µm, respectively). Exception made the GAP43 intra-sarcomere distance in stretched fibers which coincided with that of CRUs (both 1.6 µm). This discrepancy could be due to: a) a real superimposition of GAP-43 with triads in stretched fibers; or b) simply to the lower resolution of confocal microscopy compared to EM, which may result in less precise measurements.

## Discussion

Much is known about the localization and functions of GAP43 in the nervous system, but few information are available about its localization in other tissues. Our results collectively show that GAP43 is expressed in both skeletal muscle cell lines and satellite cells, as well as in isolated mouse muscle fibers.

Interestingly, data obtained from skeletal muscle cell models, suggested that during the differentiation process the protein underwent a redistribution from the predominantly nuclear localization to the cytoplasmic compartment drawing a regular double cross striation.

It is well known that during differentiation processes and functional cell activity, extracellular signal transduction mechanisms are important as much as intracellular trafficking such as nuclear-cytoplasmic bidirectional shuttling [31].

Also the 23-kDa brain acid-soluble protein (BASP1 or CAP23), belonging to the same family of GAP43 and with similar functions in the nervous system, was shown to translocate from the nucleus to the cytoplasm during caspase-induced apoptosis in non neuronal model (HeLa cells), probably modulating the transcriptional activity [32].

Also amphiphysin II (BIN1) shares common features with GAP43: a) it was found expressed at the axon initial segments in neurons, and around T-tubule in skeletal muscle fiber [33] and b) it localizes exclusively in the nucleus in proliferating C2C12 cells and at cytoplasmatic level in differentiated myotubes [34]. In addition, its gene splicing alteration or mutation has been associated with muscle weakness and T-tubule alterations of myotonic dystrophy or centronuclear myopathy, suggesting for this protein not only a role in T-tubule biogenesis, but also in pathogenetic mechanisms [35]–[37].

GAP43 was shown to be acylated modulating its hydrophilic/hydrophobic status and ability to interact with the membrane bilayer [6]. This makes the protein able to dynamically interact with the membrane systems (sarcolemmal T-tubule and/or SR) of skeletal fibers participating to membrane plasticity during muscle development and function, as shown for BIN1, involved in Ca^2+^ homeostasis and T-tubule biogenesis [38].

During in vitro differentiation of myoblasts into myotubes, an unconventional myosin heavy chain, named MYO18B, was shown to be regulated. In particular, immuno-localization of MYO18B protein in skeletal muscle cells showed that this protein was located in the cytoplasm of undifferentiated myoblasts, while, a fraction of this protein is accumulated in a subset of nuclei in differentiated myotubes [39]. This suggested different regulatory roles for MYO18B in the myogenesis.

Interestingly, it has also been demonstrated that calmodulin-associated serine/threonine kinase (CASK) is localized in the nucleus in undifferentiated myoblasts, but it is predominantly in the cytoplasm in differentiated myotubes of the C2C12 cell line. Considering that this protein was also localized on post-synaptic membranes of the neuromuscular junction, at least two roles have been hypothesized for this protein: 1) a transcriptional co-activator or 2) structural component in skeletal muscle cells [40].

Even thought GAP43 functions in skeletal muscle models remain to be elucidated, it could be postulated that GAP43 translocation may represent a regulatory process or a structural reorganization linked to the differentiation of skeletal muscle phenotype.

The presence of GAP43 in skeletal muscle tissue was also reported by other Authors. Heuss and Colleagues showed an increased immuno-reactivity for the protein in some human biopsies, of different muscle types, deriving from patients suffering myopathies [18]. In the same study, immuno-cytochemistry experiments revealed that GAP43 appeared in some, but not in all, myopathic biopsies. In particular, the protein immuno-reactivity appeared randomly distributed over the nuclear and perinuclear area of regenerating muscle fibers and on the inner face of the sarcolemma in hypotrophic type I fibers in congenital fiber type disproportion [18], [21]. Using EM technique and immuno-gold labeling for GAP43, the same group reported that GAP43 was specifically observed both in the nuclei and perinuclear cytoplasm of myoblast-like cells, but not in nuclei and myofibrils in muscle fibers from human muscle biopsies of patients with idiopathic polymyosistis [19]. For these reasons, these Authors hypothesized that GAP43 could be differentially expressed in regenerating mature fibers and developmentally regulated in mature myofibers participating in cytoplasmatic and transmenbrane signal transduction [18], [19], [21]. These observations correlate well with our findings on GAP43 localization in *in vitro* muscle cell models and in wild type mouse EDL cross sections. In the former, GAP43 localization was dependent on the cell differentiation status, in EDL fibers the protein presented a reticular structure surrounding the myofibrils close but not co-localizing with mitochondria.

However, a possible limit of the studies mentioned above, could be the assay of the presence of the protein only in cross-sections of muscle biopsies using immuno-histochemistry at low magnification, losing the pattern of the functional structures of the fibers.

**Figure 8:**
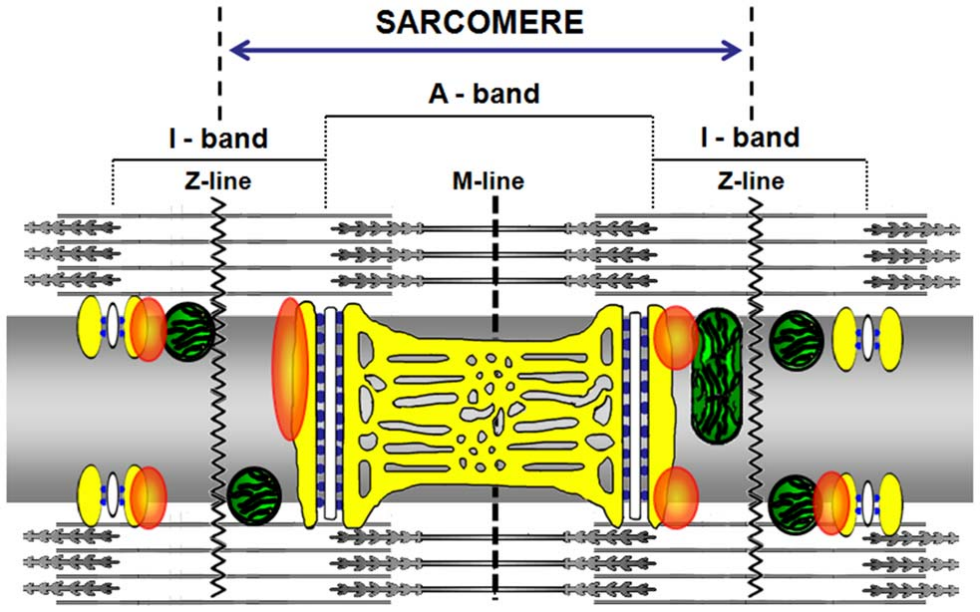
Model proposing GAP43 positioning in skeletal muscle. In the proposed model the fiber is seen along its longitudinal axis (as in figure 3). The SR is reproduced in yellow, while mitochondria on both sides of the Z–lines, are represented in green. The white structures represent the T-tubules and the blue spots are RYRs. Orange blobs represent GAP43.

Our results obtained in EDL fibers revealed that the GAP43 showed a regular double striations running parallel with CRUs and mitochondria. Qualitative and quantitative analyses of experiments performed in immuno-fluorescence and EM on resting and stretched fibers (see Figures 5, 6, 7) allowed to design and propose the model shown in Figure 8 depicting the possible GAP43 localization. In muscle fibers, GAP43 (orange blobs in Figure 8) was present in the I-band between CRUs (white structures/blue spots in Figure 8) and mitochondria but closer to the triads than to mitochondria.

Interestingly, GAP43 structure is characterized by an IQ domain and a neuromodulin domain [3]. The GAP43 IQ domain binds calmodulin (CaM) and the affinity of CaM to GAP43 is independent on Ca^2+^ concentration. In addition, the phosphorylation of GAP-43 on ser41, prevents CaM binding. For these reasons, in neurons, it has been proposed that GAP43 could serve as a CaM “sponge”, sequestering CaM at sites near membrane and releasing it in response to second messengers that result in GAP43 phosphorylation [41], [42].

Recently it has been demonstrated that CaM modulates RyR1 when cytosolic [Ca^2+^] increases during repetitive or prolonged fiber depolarization. In these conditions endogenous Ca-CaM contributes to the inactivation of Ca^2+^ release trough ryanodine receptors [43]. The CaM regulatory effect is not restricted to RYR receptors. Stroffekova demonstrated by FRET and patch-clamp experiments. that CaM binds the IQ domain of skeletal muscle L-type Ca^2+^ channel (Cav1.1) promoting the inactivation of the channel [44].

Considering the interaction between GAP43 and CaM, it could be speculated that GAP43, interacting with CRUs and being near mitochondria, plays a key role in participating to the dynamic handling of intracellular Ca^2+^.

It is of note that regulatory role of CaM in skeletal muscle is not confined to the modulation of DHPR/RYR system but is also involved in calcineurin/NFAT or Ca^2+^/calmodulin-dependent kinase (CaMKs) signaling pathways linked to the activation of several transcriptional factors that regulate expression of hypertrophic genes, mitochondrial biogenesis and oxidative genes [45], [46].

All these evidences together with the peculiar localization of the GAP43 between CRUs and mitochondria and its ability to bind CaM, support possible physiological roles for this protein in muscle development, as well as muscle contractile and metabolic properties.

This work leads the way to further investigation of the possible physiological and structural role of GAP43 protein in adult fiber function and disease.

## Supporting Information

Figure S1 **GAP43 detection using a different antibody (HPA-GAP43) shows similar immuno-reactions both in Western blot and confocal images as that described using the mGAP43 antibody.** A). Western blot of protein homogenates deriving from proliferating myoblasts (satellite), differentiated myotubes (Myotubes), mouse Extensor Digitorum Longus (EDL) and mouse brain (Brain, used as positive control). Membrane probed with rabbit polyclonal anti-GAP43 (HPA-GAP43) shows for all samples approximately the same immuno-reactions at ∼43 kDa as with the mGAP43 antibody. B) Immuno-fluorescence of EDL fiber using HPA-GAP43 antibody shows the same regular localization observed with mGAP43 antibody even with small differences (punctuate pattern instead of a double cross striation). Bars: 10 µm.
(TIF)

Figure S2 **GAP43 localization in other cell lines (C2C12 and L6E9) before and after differentiation is similar to that described in muscle cells (see** **Figure 2** **).** Immuno-fluorescence of proliferating (A and C) and differentiated (B and D) C2C12 and L6E9 cells reveals changes in localization of GAP43 during differentiation, as already shown in myoblasts and myotubes (see Figure 2): GAP43 is mainly localized in the nucleus in un-differentiated cells, while displays a sarcomeric pattern in differentiating myotubes. Bars: A-D, 10 µm.
(TIF)
