# Supplementary figures and images for: Growth Associated Protein 43 Is Expressed in Skeletal Muscle Fibers and Is Localized in Proximity of Mitochondria and Calcium Release Units
Source: PLoS One. 2013 Jan 7;8(1):e53267. doi: 10.1371/journal.pone.0053267 (PMC3538766; doi:10.1371/journal.pone.0053267)

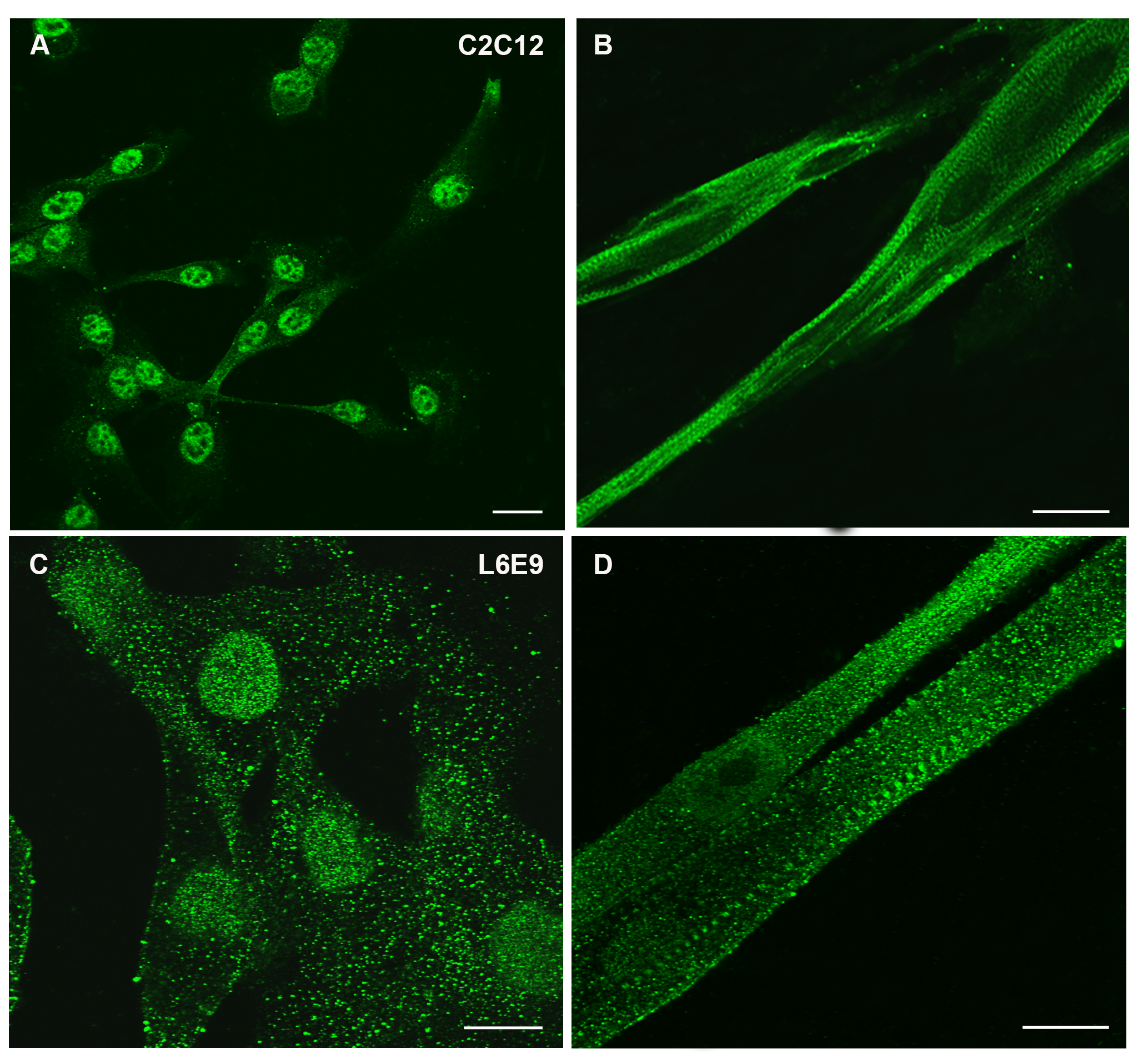

Supplement: Figure S1 — GAP43 detection using a different antibody (HPA-GAP43) shows similar immuno-reactions both in Western blot and confocal images as that described using the mGAP43 antibody. A). Western blot of protein homogenates deriving from proliferating myoblasts (satellite), differentiated myotubes (Myotubes), mouse Extensor Digitorum Longus (EDL) and mouse brain (Brain, used as positive control). Membrane probed with rabbit polyclonal anti-GAP43 (HPA-GAP43) shows for all samples approximately the same immuno-reactions at ∼43 kDa as with the mGAP43 antibody. B) Immuno-fluorescence of EDL fiber using HPA-GAP43 antibody shows the same regular localization observed with mGAP43 antibody even with small differences (punctuate pattern instead of a double cross striation). Bars: 10 µm. (TIF) [file pone.0053267.s001.tif]

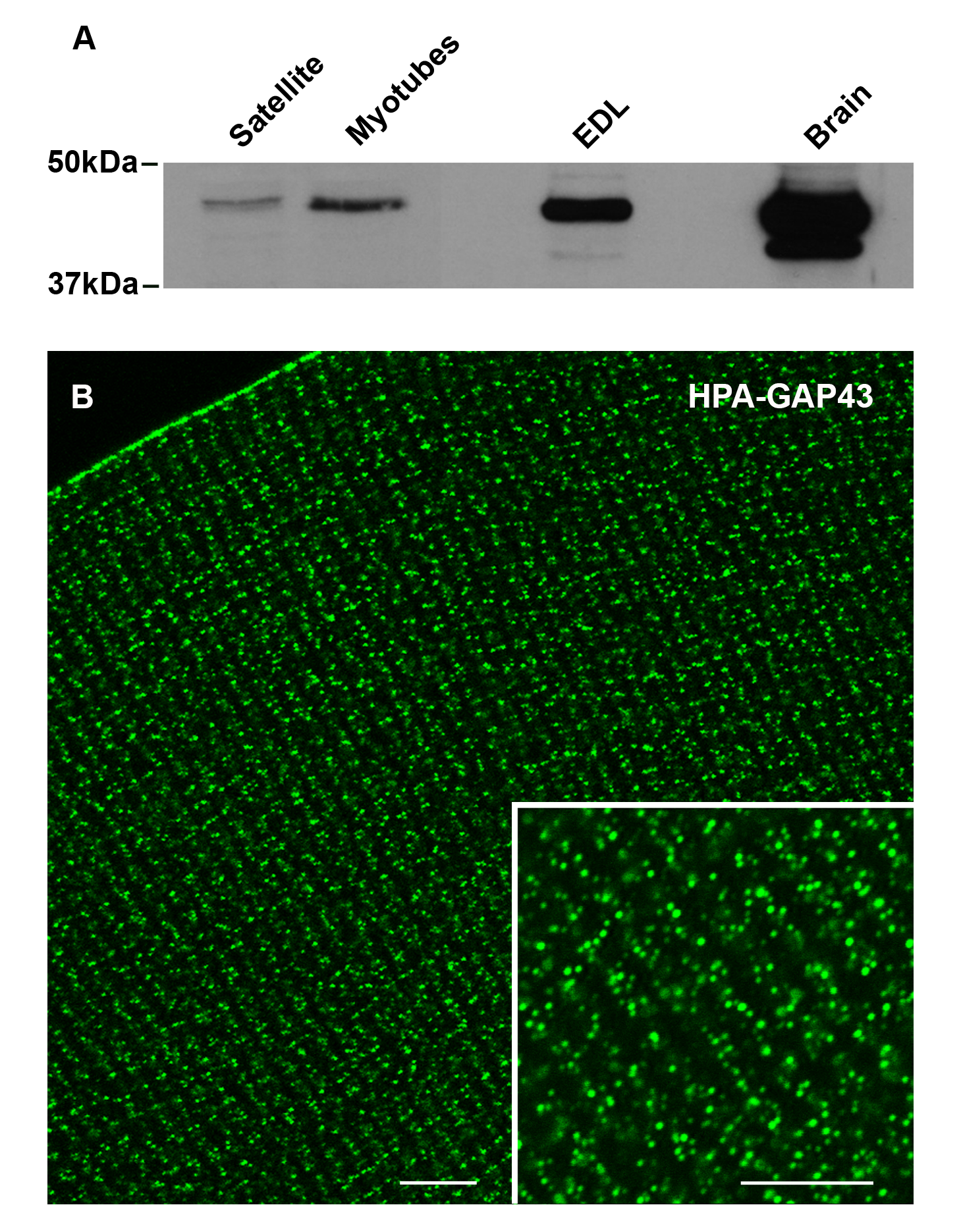

Supplement: Figure S2 — GAP43 localization in other cell lines (C2C12 and L6E9) before and after differentiation is similar to that described in muscle cells (see Figure 2 ). Immuno-fluorescence of proliferating (A and C) and differentiated (B and D) C2C12 and L6E9 cells reveals changes in localization of GAP43 during differentiation, as already shown in myoblasts and myotubes (see Figure 2): GAP43 is mainly localized in the nucleus in un-differentiated cells, while displays a sarcomeric pattern in differentiating myotubes. Bars: A-D, 10 µm. (TIF) [file pone.0053267.s002.tif]
